# Dermatofibrosarcoma Protuberans in Children: Favorable Outcomes Using Wide Local Excision

**DOI:** 10.1111/pde.70087

**Published:** 2025-12-01

**Authors:** Jawad Aqeel, Claire E. Holtz, Grace A. Osborne, Steven J. Kasten, Kelly L. Harms, Elisabeth A. Pedersen

**Affiliations:** ^1^ Department of Dermatology, Division of Cutaneous Surgery and Oncology University of Michigan Medical School Ann Arbor Michigan USA; ^2^ College of Literature, Science, and the Arts University of Michigan Ann Arbor Michigan USA; ^3^ Department of Dermatology, Division of Pediatric Dermatology University of Michigan Medical School Ann Arbor Michigan USA; ^4^ Department of Plastic Surgery University of Michigan Medical School Ann Arbor Michigan USA

**Keywords:** dermatofibrosarcoma protuberans, margins, pediatric dermatology, wide local excision

## Abstract

**Background:**

Dermatofibrosarcoma protuberans (DFSP) is a rare and locally aggressive cutaneous sarcoma. Surgical excision remains first‐line therapy, including for pediatric patients. However, given the rarity of DFSP, specific treatment recommendations for children have not been well defined. To inform pediatric‐specific management, we analyzed outcomes in a retrospective cohort of pediatric DFSP patients treated with wide local excision (WLE).

**Methods:**

A single‐center retrospective review of clinical records from 2004 through 2024 was conducted evaluating patients < 20 years of age who were diagnosed with DFSP and underwent treatment with WLE. Patients were divided into two groups based on treatment with relatively narrow (< 2 cm) or standard margins (≥ 2 cm). Descriptive analyses were performed.

**Results:**

A total of 17 pediatric DFSP patients underwent WLE. The mean surgical margin was 1.76 ± 0.54 cm, and 14 of 16 evaluable patients (87.5%) were treated with margins of 2 cm or less. Complete excision after one surgery was achieved in 76.5% of patients. No patients experienced local or distant recurrence, and none required adjuvant therapy. Interestingly, 64.7% (11/17) had documentation describing the lesion being first noted at or around the time of birth. For patients with over 60 months of follow‐up, the 5‐year recurrence‐free survival, disease‐specific survival, and overall survival were all 100%.

**Conclusions:**

When appropriately selected and performed, WLE with a 1–2 cm initial margin, followed by re‐excision when needed to achieve clear margins, does not compromise survival or risk of recurrence and may be considered an effective treatment option in pediatric DFSP.

## 1 Introduction

Dermatofibrosarcoma protuberans (DFSP) is a rare cutaneous sarcoma that is locally aggressive with a high propensity for recurrence if not completely excised [1, 2, 3]. It is typically associated with characteristic chromosomal translocations, the most common of which is *t*(17:22) encoding the *COL1A1::PDGFB* fusion protein, although other fusions have been described [4, 5, 6]. DFSP has a peak incidence among patients aged 30–49 years, but can present at any age, including children [7]. Clinically, DFSP presents as a slow‐growing plaque or nodule enlarging over months to years. Due to its rarity and resemblance to more common cutaneous lesions, such as keloids and cysts, DFSP may develop unrecognized and become large if left untreated. While metastasis is rare, delayed treatment or the presence of fibrosarcomatous change on histopathology may portend more aggressive disease. The incidence of DFSP is estimated to be 6.25 cases per million person‐years in the general population, but is even more rare in the pediatric population with an estimated incidence of 1.22 cases per million person‐years [7]. This rarity further exacerbates diagnostic delays in children and has contributed to a lack of data with regards to pediatric‐specific management of DFSP. Studies have reported that DFSP may be noticed initially as a congenital or childhood lesion, but may not be diagnosed until adulthood, suggesting that development of DFSP during childhood may be underestimated [8, 9].

Current treatment guidelines for DFSP are based on adult patients. The National Comprehensive Cancer Network (NCCN) recommends Mohs micrographic surgery (MMS), other forms of peripheral and deep en face margin assessment (PDEMA), or wide local excision (WLE), if PDEMA is not available [10]. For advanced or unresectable disease, systemic treatment with imatinib is used [10]. In children, surgery poses unique challenges as achieving adequate margins may be challenging due to their smaller body sizes, anatomic differences from adults, and inability to tolerate treatment under local anesthetic [11]. There is limited literature specifically addressing how the prognosis, treatment, and management outcomes of DFSP differ in pediatric patients compared to adults. Small studies have suggested WLE and MMS to be effective treatments for DFSP in the pediatric population; however, the management of pediatric DFSP remains challenging and has mostly been adapted from adult patients and the literature [12, 13]. It was recently demonstrated in a small cohort that both MMS and WLE had similar cure rates for DFSP in the pediatric population, and WLE had a shorter time to treatment, perhaps in part due to logistical difficulties in coordinating MMS for pediatric patients [14]. However, the specific margins that lead to these excellent outcomes were not assessed. To address this paucity of data, we analyzed a retrospective cohort of pediatric DFSP patients treated with WLE from a single institution, with particular attention to surgical margin width and patient outcomes.

## 2 Methods

Approval for this study was obtained from the University of Michigan Medical School Institutional Review Board. A single‐center retrospective review was conducted of clinical records for all patients diagnosed with DFSP and treated from 2004 through April 2025. Patients were included if they had a histopathologic diagnosis of DFSP, were aged younger than 20 years at the time of diagnosis, and underwent treatment with WLE. Patients were excluded if they received treatment for DFSP at another institution prior to presentation or if medical records were incomplete regarding treatment and follow‐up.

Data collected included patient demographics (age, year of diagnosis, sex assigned at birth), tumor characteristics (location, size, histologic subtype, and presence or absence of fibrosarcomatous change), details of surgical treatment (excision margin size, need for re‐excision), and postoperative outcomes. Complete excision was defined as negative surgical margins on pathology after the first WLE without needing a second re‐excision procedure for positive margins. Additionally, the use of adjuvant therapies, such as imatinib or radiation, was documented. Any instances of local recurrence or metastasis that occurred during the follow‐up period were recorded. Following initial data collection, patients were divided into two groups based on whether they had a relatively narrow (< 2 cm) or standard WLE margin (≥ 2 cm). The threshold for narrow and wide surgical margins was based upon the NCCN adult DFSP management guidelines of WLE with 2–4 cm margins.

Descriptive analyses were performed to summarize patient demographics, tumor characteristics, and clinical outcomes following WLE. Continuous variables were expressed as mean ± standard deviation. Categorical variables were expressed as frequencies or percentages. All statistical analyses and calculations were performed using Microsoft Excel and Stata.

## 3 Results

### 3.1 Patient Demographics and Tumor Characteristics

After applying study criteria, a total of 17 patients under 20 years of age with DFSP who underwent WLE as their primary treatment were identified. Patient demographics, tumor characteristics, and treatment outcomes are summarized in Table 1. The mean age at the time of diagnosis was 13.2 ± 4.9 years (median: 15.0; range: 2–20). 76.5% (13/17) of the cohort was female. Interestingly, 64.7% (11/17) had documentation describing the lesion being first noted at or around the time of birth. The average clinical size of DFSP lesions at initial presentation was 2.73 ± 1.59 cm (range: 0.4–5.5 cm). The most common site of primary disease was the trunk (8/17, 47.1%), followed by the extremities (6/17, 35.3%), and then the head and neck (3/17, 17.6%). All lesions were consistent with classic or typical DFSP on histopathology; no specimen demonstrated fibrosarcomatous change. No pathology reports described the tumor as a Bednar tumor, atrophic, myxoid, or other variants. Preoperative imaging with magnetic resonance imaging (MRI) was obtained in two cases to further characterize the lesions.

**TABLE 1:** Demographics and clinical characteristics of 17 patients.

Age at diagnosis (years)	Sex	Site	Pathology	FS	Tumor size (cm)	Surgical margins (cm)	Initial WLE outcome	Re‐excision	Recurrence	Duration of follow up (months)
2	M	Scalp	DFSP	No	2	1.5	Margins free	N/a	No	50
4	F	Abdomen	DFSP	No	2	1.7	Margins free	N/a	No	1
10	F	Abdomen	DFSP	No	2.5	1.5	Margins free	N/a	No	66
10	F	Lower extremity	DFSP	No	1.5	2.5	Margins free	N/a	No	33
11	M	Chest	DFSP	No	1	1.5	Positive margin	Margins free	No	65
11	F	Lower extremity	DFSP	No	3	2	Margins free	N/a	No	13
12	M	Lower extremity	DFSP	No	0.4	1.5	Margins free	N/a	No	60
14	M	Abdomen	DFSP	No	5	1.5–2	Positive margin	Margins free	No	58
15	F	Lower extremity	DFSP	No	2	1	Margins free	N/a	No	60
15	F	Lower extremity	DFSP	No	3.2	2	Margins free	N/a	No	26
15	F	Lower extremity	DFSP	No	5.5	2	Margins free	N/a	No	7
16	F	Forehead	DFSP	No	2	2	Positive margin	Margins free	No	98
16	F	Abdomen	DFSP	No	2.5	2	Margins free	N/a	N/A	N/A
17	F	Back	DFSP	No	5.5	1	Positive margin	Margins free	No	46
18	F	Abdomen	DFSP	No	1.3	2	Margins free	N/a	No	58
19	F	Back	DFSP	No	5	3	Margins free	N/a	No	43
20	F	Neck	DFSP	No	2	1	Margins free	N/a	No	23

At the time of diagnosis, all patients were without any major comorbidities or known immunodeficiencies, with the exception of two patients who each carried a diagnosis of juvenile idiopathic arthritis (JIA). One of these patients had been treated previously with methotrexate and tocilizumab, but did not receive immune suppressive therapy at the time of diagnosis or during treatment for DFSP. The other never received any immunosuppressive therapy.

### 3.2 Treatment and Outcomes

**FIGURE 1:**
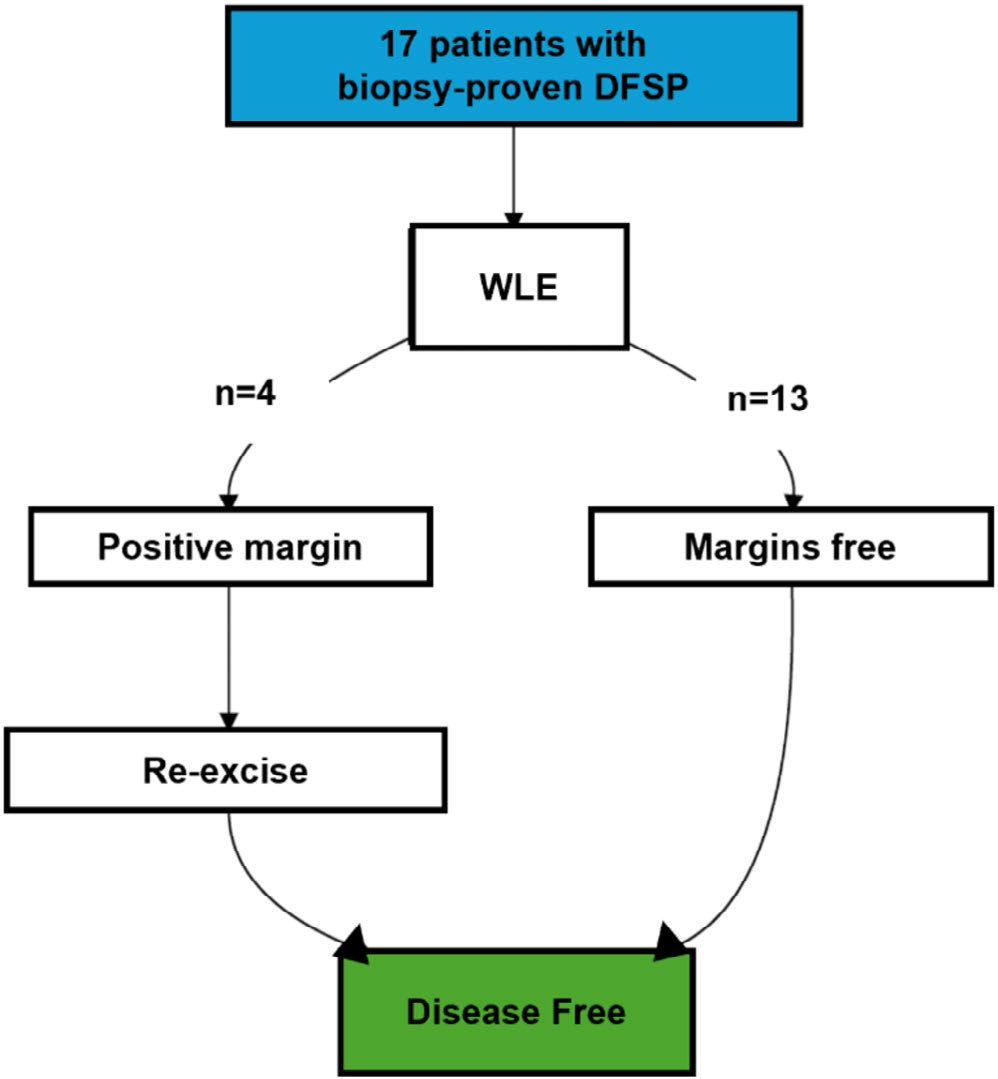
Schematic of wide local excision (WLE) treatment for pediatric patients diagnosed with DFSP.

**FIGURE 2:**
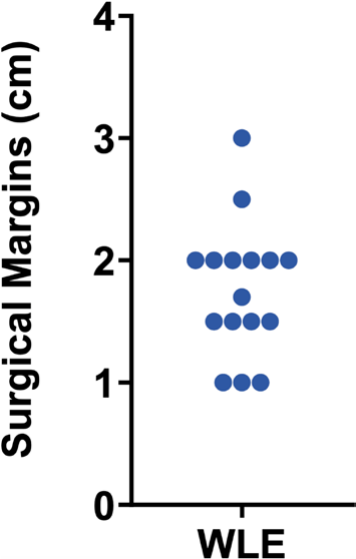
Distribution of WLE margins used in pediatric patients diagnosed with DFSP.

All 17 patients underwent WLE, as depicted in Figure 1, which outlines the management strategy for the cohort. The mean surgical margin taken during WLE was 1.76 ± 0.54 cm (range: 1.0–3.0 cm), with the distribution of margin sizes illustrated in Figure 2. Notably, the analysis of surgical margins excluded one patient whose medical records specified an imprecise margin range of “1.5–2.0 cm” precluding an exact measurement. Of the 16 remaining cases, 14 (87.5%) were treated with margins of 2 cm or less. Complete excision was achieved in 13 of the 17 patients (76.5%). All four patients with positive margins during the initial WLE underwent prompt re‐excision, and in each case demonstrated negative margins following the second re‐excision procedure. No patient required more than one re‐excision. Key data regarding clinical characteristics and overall treatment results in the cohort are summarized in Table 2.

**TABLE 2:** Summary of clinical characteristics and treatment outcomes in 17 patients.

	Mean (SD, range)
Age of diagnosis (years)	13.2 (4.9, 2–20)
Clinical size (cm)	2.7 (1.6, 0.4–5.5)
WLE margins (cm)	1.76 (0.54, 1.0–3.0)
Follow up duration (months)	44 (26, 1–98)
Female	13 (76.5)
Male	4 (23.5)

	N (% of total)
Trunk	8 (47.1)
Extremities	6 (35.3)
Head & neck	3 (17.6)

Patients were followed for an average of 45 months after excision (median: 49; range 2–99). For the seven patients with follow‐up for 59 months or more (approximating 5 years), the recurrence‐free survival, disease‐specific survival, and overall survival were all 100%. No local or distant recurrences have been observed in the cohort to date. There are no cases of metastatic disease. Additionally, none of the patients in the cohort received adjuvant therapy with targeted immunotherapy using imatinib or radiation therapy, either before or after surgery.

### 3.3 Margin Width

The margin width was then evaluated by age to determine if any trends were present based on age or disease characteristics. As shown in Table 3, eight patients (50.0%) underwent excision with margins < 2 cm, and eight patients (50.0%) underwent excision with margins ≥ 2 cm. In our cohort, patients treated with narrower margins tended to be younger than those with wide margins. Lesion location was comparable between the two groups. Initial tumor size appeared to correlate with margin size. Smaller lesions (< 2.5 cm) were more often managed with < 2 cm margins (6/8, 75.0%), while larger lesions (≥ 2.5 cm) more frequently utilized margins with ≥ 2 cm (5/8, 62.5%). 3/16 (18.8%) patients had positive margins following the WLE and required a second excision. No patients required more than two surgical stages.

**TABLE 3:** Pediatric DFSP tumor and treatment characteristics by margin size.

	Reduced margin < 2 cm (*n* = 8)	Standard margin ≥ 2 cm (*n* = 8)	Total (*n* = 16)
Age at diagnosis			
Median (range)	11.5 (2–20)	15.5 (10–19)	15.0 (2–20)
Primary site			
Head & neck	2 (25.0%)	1 (12.5%)	3 (18.8%)
Trunk	4 (50.0%)	3 (37.5%)	7 (43.8%)
Extremities	2 (25.0%)	4 (50.0%)	6 (37.5%)
Tumor size			
< 2.5 cm	6 (75.0%)	3 (37.5%)	9 (56.3%)
≥ 2.5 cm	2 (25.0%)	5 (62.5%)	7 (43.8%)
Required second excision			
No	6 (75.0%)	7 (87.5%)	13 (81.3%)
Yes	2 (25.0%)	1 (12.5%)	3 (18.8%)
Adjuvant therapy			
Yes	0 (0.0%)	0 (0.0%)	0 (0.0%)
No	8 (100%)	8 (100%)	16 (100%)
Disease recurrence			
Yes	0 (0.0%)	0 (0.0%)	0 (0.0%)
No	8 (100%)	8 (100%)	16 (100%)

## 4 Discussion

For patients with DFSP, surgical excision remains first‐line therapy, including for pediatric patients. Current NCCN guidelines recommend excision with MMS, other forms of deep and en face peripheral margin assessment, or WLE [10]. However, given the rarity of DFSP in this population, specific treatment recommendations and outcomes for children have not been well studied. Here, we describe one of the largest cohorts of pediatric DFSP patients treated in the United States in which we evaluated the details of surgical treatment and subsequent outcomes. In this cohort, we found that pediatric patients tended to present with small primary tumors (< 3 cm) and underwent WLE with margins less than or equal to 2 cm. In our cohort, most margins were between 1.5 and 2.0 cm. Following WLE, all patients experienced excellent outcomes without evidence of local or distant recurrence or need for adjuvant treatment. No mortalities were observed. Basta et al. similarly found that narrower margins were effective for pediatric patients with melanoma, suggesting that conservative margins may be plausible when treating cutaneous oncologic lesions in pediatric patients [15].

Our finding of 100% survival of pediatric patients with DFSP is similar to recent studies including a population‐based study of 451 patients with DFSP in the United States, and a similar‐sized cohort that included patients treated with WLE and MMS [14, 16]. Regarding the risk of local recurrence, our cohort of 17 patients reveals that all patients had no evidence of recurrence, which appears improved from studies from our country and others with recurrence rates ranging from 13% to 21% in mostly similarly sized cohorts [17, 18, 19]. It is encouraging that our cohort validates the findings that pediatric patients who achieve clear margins typically have an excellent prognosis. Interestingly, over half of the patients in our cohort had initial lesions noted at the time of birth or early infancy, some with disease likely present for over a decade, and fortunately still experienced excellent outcomes.

When DFSP presents in pediatric patients, questions regarding surgical planning will arise. In a recent cohort study by Brough et al., all patients aged less than 10 treated for DFSP with MMS required general anesthesia [20]. In this cohort, the number of MMS layers to clear the tumor ranged from one to five with an average of two layers per patient, with general anesthesia required for each layer and closure [20]. Given that pediatric patients may be unable to tolerate an extensive procedure and reconstruction under local anesthesia, minimizing the logistics of surgical intervention and anesthesia with conservative WLE may provide a similarly favorable and effective option [14]. Less than 25% of patients in our cohort (4/17, 23.5%) required a second procedure, thus making WLE a reasonable option in this regard.

Taken together, our findings reveal that pediatric patients with DFSP have an excellent prognosis following surgical treatment with clear margins. When appropriately selected and performed, WLE with relatively narrow reduced margins (1–2 cm) does not compromise survival or risk of recurrence and may be considered an effective treatment option in this population.

## Conflicts of Interest

The authors declare no conflicts of interest.

## Data Availability

Data sharing not applicable to this article as no datasets were generated or analyzed during the current study.

## References

[1] Y. J. Tsai , P. Y. Lin , K. Y. Chew , and Y. C. Chiang , “Dermatofibrosarcoma Protuberans in Children and Adolescents: Clinical Presentation, Histology, Treatment, and Review of the Literature,” Journal of Plastic, Reconstructive & Aesthetic Surgery 67, no. 9 (2014): 1222–1229. 10.1016/j.bjps.2014.05.031 24973861

[2] R. C. Fields , M. Hameed , L. X. Qin , et al., “Dermatofibrosarcoma Protuberans (DFSP): Predictors of Recurrence and the Use of Systemic Therapy,” Annals of Surgical Oncology 18, no. 2 (2011): 328–336. 20844969 10.1245/s10434-010-1316-5 PMC4310211

[3] K. Harati , K. Lange , O. Goertz , et al., “A Single‐Institutional Review of 68 Patients With Dermatofibrosarcoma Protuberans: Wide Re‐Excision After Inadequate Previous Surgery Results in a High Rate of Local Control,” World Journal of Surgical Oncology 15, no. 1 (2017): 5. 28056985 10.1186/s12957-016-1075-2 PMC5217543

[4] I. Nakamura , Y. Kariya , E. Okada , et al., “A Novel Chromosomal Translocation Associated With COL1A2‐PDGFB Gene Fusion in Dermatofibrosarcoma Protuberans: PDGF Expression as a New Diagnostic Tool,” JAMA Dermatology 151, no. 12 (2015): 1330–1337. 26332510 10.1001/jamadermatol.2015.2389

[5] M. P. Simon , F. Pedeutour , N. Sirvent , et al., “Deregulation of the Platelet‐Derived Growth Factor B‐Chain Gene via Fusion With Collagen Gene COL1A1 in Dermatofibrosarcoma Protuberans and Giant‐Cell Fibroblastoma,” Nature Genetics 15, no. 1 (1997): 95–98. 8988177 10.1038/ng0197-95

[6] N. Sirvent , G. Maire , and F. Pedeutour , “Genetics of Dermatofibrosarcoma Protuberans Family of Tumors: From Ring Chromosomes to Tyrosine Kinase Inhibitor Treatment,” Genes, Chromosomes & Cancer 37, no. 1 (2003): 1–19. 12661001 10.1002/gcc.10202

[7] J. Maghfour , X. Genelin , J. Olson , A. Wang , L. Schultz , and T. W. Blalock , “The Epidemiology of Dermatofibrosarcoma Protuberans Incidence, Metastasis, and Death Among Various Population Groups: A Surveillance, Epidemiology, and End Results Database Analysis,” Journal of the American Academy of Dermatology 91, no. 5 (2024): 826–833. 38908718 10.1016/j.jaad.2024.05.088

[8] L. Martin , P. Combemale , M. Dupin , et al., “The Atrophic Variant of Dermatofibrosarcoma Protuberans in Childhood: A Report of Six Cases,” British Journal of Dermatology 139, no. 4 (1998): 719–725. 10025975

[9] R. M. Strauss , W. J. Merchant , P. Roberts , A. L. Wright , and S. M. Clark , “A Case of Childhood Dermatofibrosarcoma Protuberans Without Detected Cytogenetic Abnormality,” British Journal of Dermatology 148, no. 5 (2003): 1051–1055. 12786842 10.1046/j.1365-2133.2003.05339.x

[10] J. Bordeaux , R. Blitzblau , S. Z. Aasi , et al., “Dermatofibrosarcoma Protuberans, Version 1.2025, NCCN Clinical Practice Guidelines in Oncology,” Journal of the National Comprehensive Cancer Network 23, no. 1 (2025): e250001. 39819674 10.6004/jnccn.2025.0001

[11] H. Zargham and A. Khachemoune , “Systematic Review of Mohs Micrographic Surgery in Children: Identifying Challenges and Practical Considerations for Successful Application,” Journal of the American Academy of Dermatology 85, no. 1 (2021): 152–161. 33011324 10.1016/j.jaad.2020.09.052

[12] R. I. Kornik , L. K. Muchard , and J. M. Teng , “Dermatofibrosarcoma Protuberans in Children: An Update on the Diagnosis and Treatment,” Pediatric Dermatology 29, no. 6 (2012): 707–713. 22780227 10.1111/j.1525-1470.2012.01767.x

[13] J. R. Marcus , J. W. Few , C. Senger , and M. Reynolds , “Dermatofibrosarcoma Protuberans and the Bednar Tumor: Treatment in the Pediatric Population,” Journal of Pediatric Surgery 33, no. 12 (1998): 1811–1814. 9869058 10.1016/s0022-3468(98)90292-5

[14] M. C. Caussade , C. Y. Huang , G. Stockton Hogrogian , J. R. Treat , N. J. Balamuth , and A. C. Yan , “Pediatric Dermatofibrosarcoma Protuberans: Surgery Outcomes,” Pediatric Dermatology 42 (2025): 1027–1029, 10.1111/pde.15942. 40254463

[15] A. V. Basta , C. D. Fritz , Y. J. Chiang , et al., “The Impact of Surgical Margin in Wide Local Excision of Pediatric Melanoma—An Argument for a More Conservative Approach,” Journal of Pediatric Surgery 60, no. 1 (2025): 161897. 39349345 10.1016/j.jpedsurg.2024.161897

[16] G. A. Rubio , A. Alvarado , D. J. Gerth , J. Tashiro , and S. R. Thaller , “Incidence and Outcomes of Dermatofibrosarcoma Protuberans in the US Pediatric Population,” Journal of Craniofacial Surgery 28, no. 1 (2017): 182–184. 27922973 10.1097/SCS.0000000000003203

[17] C. W. Iqbal , S. St Peter , and M. B. Ishitani , “Pediatric Dermatofibrosarcoma Protuberans: Multi‐Institutional Outcomes,” Journal of Surgical Research 170, no. 1 (2011): 69–72. 21429521 10.1016/j.jss.2011.01.042

[18] C. J. Posso‐De Los Rios , I. Lara‐Corrales , and N. Ho , “Dermatofibrosarcoma Protuberans in Pediatric Patients: A Report of 17 Cases,” Journal of Cutaneous Medicine and Surgery 18, no. 3 (2014): 180–185. 24800706 10.2310/7750.2013.13099

[19] Z. Zhang , Y. Lu , C. Shi , M. Chen , X. He , and H. Zhang , “Pediatric Dermatofibrosarcoma Protuberans: A Clinicopathologic and Genetic Analysis of 66 Cases in the Largest Institution in Southwest China,” Frontiers in Oncology 13 (2023): 1017154. 36776313 10.3389/fonc.2023.1017154 PMC9916051

[20] K. R. Brough , M. J. Youssef , D. S. Winchester , C. L. Baum , B. A. Sharaf , and R. K. Roenigk , “Mohs Micrographic Surgery for Dermatofibrosarcoma Protuberans in 7 Patients Aged 10 Years and Younger,” Journal of the American Academy of Dermatology 86, no. 6 (2022): 1429–1431. 34214620 10.1016/j.jaad.2021.06.856

